# Benchmarking large language models for personalized, biomarker-based health intervention recommendations

**DOI:** 10.1038/s41746-025-01996-2

**Published:** 2025-10-27

**Authors:** Hans Jarchow, Christoph Bobrowski, Steffi Falk, Andreas Hermann, Anton Kulaga, Johann-Christian Põder, Maximilian Unfried, Nikolay Usanov, Bijan Zendeh, Brian K. Kennedy, Sebastian Lobentanzer, Georg Fuellen

**Affiliations:** 1 Institute for Biostatistics and Informatics in Medicine and Ageing Research, Rostock University Medical Center, Rostock, Germany; 2 Klinik für Neurologie und Geriatrie, Johanniter-Krankenhaus Stendal, Stendal, Germany; 3 Klinik für Unfall-, Hand- und Wiederherstellungschirurgie, Rostock University Medical Center, Rostock, Germany; 4 Translational Neurodegeneration Section “Albrecht Kossel”, and Rostock University Medical Center, Rostock, Germany; 5 German Center for Neurodegenerative Diseases (DZNE), Rostock/Greifswald, Rostock, Germany; 6 Ethics in Theology and Medicine, Faculty of Theology, Rostock University Faculty of Theology, Rostock, Germany; 7 Healthy Longevity Translational Research Program, Yong Loo Lin School of Medicine, National University of Singapore, Singapore, Singapore; 8 Department of Biochemistry, Yong Loo Lin School of Medicine, National University of Singapore, Singapore, Singapore; 9 HEAlthy Life Extension Society (HEALES), Brussels, Belgium; 10 Dept. of Neurology, Rostock University Medical Center, Rostock, Germany; 11 Department of Physiology, Yong Loo Lin School of Medicine, National University of Singapore, Singapore, Singapore; 12 Institute of Computational Biology, Computational Health Center, Helmholtz Center, Munich, Germany; 13 Open Targets, European Bioinformatics Institute, Hinxton, Cambridge UK; 14 UCD Conway Institute of Biomolecular and Biomedical Research, School of Medicine, University College Dublin, Dublin, Ireland

**Keywords:** Biomarkers, Medical research

## Abstract

The use of large language models (LLMs) in clinical diagnostics and intervention planning is expanding, yet their utility for personalized recommendations for longevity interventions remains opaque. We extended the BioChatter framework to benchmark LLMs’ ability to generate personalized longevity intervention recommendations based on biomarker profiles while adhering to key medical validation requirements. Using 25 individual profiles across three different age groups, we generated 1000 diverse test cases covering interventions such as caloric restriction, fasting and supplements. Evaluating 56000 model responses via an LLM-as-a-Judge system with clinician validated ground truths, we found that proprietary models outperformed open-source models especially in comprehensiveness. However, even with Retrieval-Augmented Generation (RAG), all models exhibited limitations in addressing key medical validation requirements, prompt stability, and handling age-related biases. Our findings highlight limited suitability of LLMs for unsupervised longevity intervention recommendations. Our open-source framework offers a foundation for advancing AI benchmarking in various medical contexts.

## Introduction

LLMs are rapidly being integrated into various aspects of medical practice and research as valuable tools in diagnostics, clinical decision making, clinical support, medical writing, education, and personalized medicine^1–4^. In geroscience and longevity medicine^5^, LLM technologies have, for example, been used for health monitoring, geriatric assessment and care, psychiatry, and risk assessment; other studies highlight the potential of these and related technologies - such as robotics - more generally, in supporting cognitive health, social interaction, assisted living, and rehabilitation^6–10^.

Benchmarks for evaluating LLMs have become indispensable to meet the rigorous standards and professionalism required in healthcare and medical research. Existing public benchmarks^11–14^ focus on assessing LLM performance in general medical and biomedical tasks, primarily using multiple-choice formats. Other datasets assess proficiency in understanding and summarizing medical texts or in disease recognition, relation extraction, and bias recognition^15–20^. Only a few benchmarks address medical interventions or treatment recommendations^21,22^, but these focus on disease-targeting interventions, and, also, not on free-text responses. A major cause of judgement bias is benchmark “contamination”, that is availability of (parts of) the benchmark data to LLMs, in their training data or while searching the internet, rendering novel data specifically valuable.

Our benchmark, reviewed and approved by physicians as domain experts, was generated de-novo and consists of 25 synthetic medical profiles (test items), each simulating a user seeking advice regarding well-known longevity interventions; we excluded interventions with only preliminary evidence of their safety and efficacy. Each test item is presented as an open query. All items consist of multiple modules that can be combined to introduce diversity in *syntax*, resulting in 1000 different test cases. To introduce *semantic* variance in the input, items were varied across two dimensions: according to age groups of individuals and types of interventions. Furthermore, we examined the impact of additional augmented context on LLM performance using Retrieval-Augmented Generation (RAG).

Both proprietary and open-source LLMs were evaluated across 5 validation requirements, using the LLM-as-a-judge paradigm^23,24^: Comprehensiveness (Comprh), Correctness (Correct), Usefulness (Useful), Interpretability/Explainability (Explnb) and Consideration of Toxicity/Safety (Safe). The LLM-as-a-judge was provided with expert commentaries, describing what we believe a good response should entail. Overall, we found that LLMs did not address all requirements equally well. However, instructing models with the requirements induced a moderate increase in model performance, confirming our perspective from last year^25^. Our results show alignment with studies that assessed similar axes of model performance, such as the work by Zakka et al.^26^, but are based on a statistically powered set of evaluations specifically focused on the domain of longevity medicine and geroscience. We developed a framework that automates LLM-based judgment, considering test-item-specific human-approved ground truths, and integrated it into BioChatter^27^. The framework is freely available at https://github.com/biocypher/biochatter and may be used and adapted for future LLM studies.

## Results

**Fig. 1:**
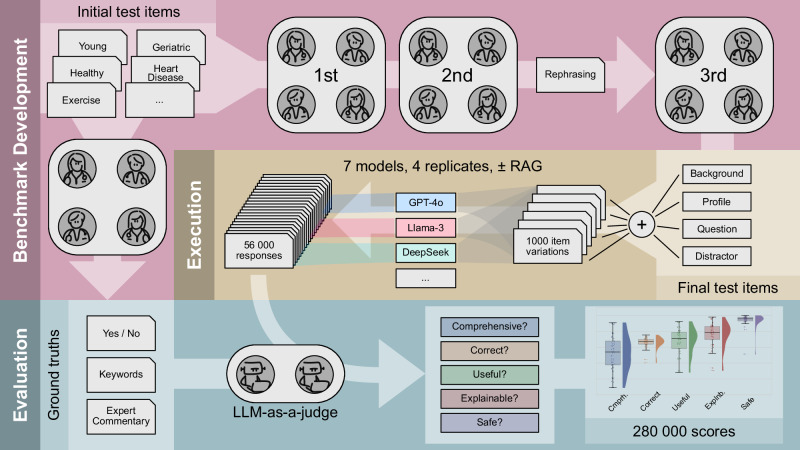
Overview of Benchmark generation and Model Evaluation procedures. The benchmark containing the test items was reviewed by physicians in two initial rounds, providing an expert commentary of expected ground truths. Subsequently, the test item components were rephrased to generate additional presentation formats. The final test item status was achieved after a third round of review, after which the test items were integrated into the test framework. 56000 LLM responses were collected and judged by LLMs based on the 5 validation requirements; the LLM acting as a judge was informed about the ground truths.

The models we evaluated for advice quality on longevity interventions included Llama 3.2 3B, Qwen 2.5 14B, DeepSeek R1 Distill Llama 70B (DSR Llama 70B), GPT-4o mini, o3 mini, GPT-4o, and the (bio)medical fine-tuned model Llama3 Med42 8B. Model responses were evaluated by GPT-4o mini, serving as the LLM-as-a-judge. For further details on model configuration as well as the selection and implementation of the judge, we refer to the “Models” section in Methods. The LLMs were tested across five system prompts of varying complexity (“System prompts” in Methods), different age groups and comorbidities of the individuals presented in the benchmark test items, as well as with and without RAG (“Domain background and Retrieval-Augmented Generation (RAG)” in Methods). The “Benchmark dataset and test items / user prompts” sections in Methods and Fig. 1 summarize the development of the benchmark.

### Accuracy of LLM responses varies significantly with validation requirements

**Fig. 2:**
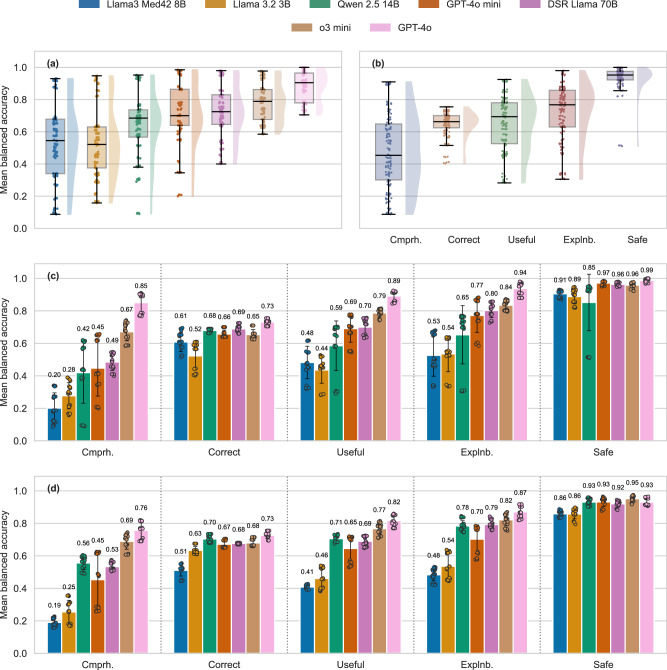
LLM mean balanced accuracy across validation requirements. We tested the models on a diverse set of test cases (*n* = 1000) multiple times (*n* = 4) across five validation requirements, comparing their performance both among models and between individual requirements. Our analysis revealed significant differences in mean balanced accuracy between requirements (*P* < 0.001). Performance is most stable for Correctness; nearly all models performed worst in Comprehensiveness (Cmprh.; *P* < 0.001) and best in Consideration of Toxicity (Safety). **a** Overall final scores of the models aggregated across all validation requirements. **b** Performance for the validation requirements across all models. **c** Mean balanced accuracy of each model per validation requirement without the application of RAG. **d** Mean balanced accuracy with the application of RAG. *The mean balanced accuracy is displayed above each bar. Error bars and individual data points (one per system prompt/replicate) are shown*.

Across validation requirements and models, GPT-4o achieved the highest overall balanced accuracy, while Llama 3.2 3B obtained the lowest (Fig. 2a). Model responses were generally considered safe, but not very comprehensive (Fig. 2b). Except for being safe, Llama 3.2 3B performed significantly worse than all other non-finetuned models (*P* < *0.001*), and GPT-4o produced responses that were significantly more comprehensive, correct, useful, interpretable and explainable (*P* < *0.001*) (Fig. 2c, Table 1). The effect of RAG was not consistent, as open-source models tended to benefit while proprietary ones tended to deteriorate (Fig. 2d, Table 1). We also evaluated Llama3 Med42 8B, a (bio)medical fine-tuned model. Its responses were significantly less comprehensive than those of all other models in the naive setting (without RAG, *P* < *0.001)*. Although it outperformed or matched Llama 3.2 3B on the remaining validation requirements, it still fell short of the other tested models.

**Table 1:** Mean balanced accuracy of models across validation requirements without (w/o) and with RAG

Evaluated Models	Comprh.			Correct			Useful			Explnb.			Safe		
	w/o RAG	RAG	$\Delta$RAG	w/o RAG	RAG	$\Delta$RAG	w/o RAG	RAG	$\Delta$RAG	w/o RAG	RAG	$\Delta$RAG	w/o RAG	RAG	$\Delta$RAG
Llama 3.2 3B	0.28 ± 0.08	0.25 ± 0.07	−0.03	0.52 ± 0.08	0.63 ± 0.02	**+0.11**	0.44 ± 0.08	0.46 ± 0.06	+0.02	0.54 ± 0.11	0.54 ± 0.08	±0	0.89 ± 0.05	0.86 ± 0.03	−0.03
Qwen 2.5 14B	0.42 ± 0.19	0.56 ± 0.04	**+0.14**	0.68 ± 0.01	0.70 ± 0.02	+0.02	0.59 ± 0.15	0.71 ± 0.02	**+0.12**	0.65 ± 0.18	0.78 ± 0.03	**+0.13**	0.85 ± 0.17	**0.93** ± 0.02	**+0.08**
DSR Llama 70B	0.49 ± 0.05	0.53 ± 0.02	+0.04	0.69 ± 0.02	0.68 ± 0.01	−0.01	0.70 ± 0.05	0.69 ± 0.03	−0.01	0.80 ± 0.04	0.79 ± 0.02	−0.01	0.96 ± 0.01	0.92 ± 0.02	−0.04
GPT-4o	**0.85** ± 0.06	**0.76** ± 0.06	−0.09	**0.73** ± 0.02	**0.73** ± 0.02	±0	**0.89** ± 0.03	**0.82** ± 0.03	−0.07	**0.94** ± 0.04	**0.87** ± 0.04	−0.07	**0.99** ± 0.01	**0.93** ± 0.02	−0.06
GPT-4o mini	0.45 ± 0.17	0.45 ± 0.16	±0	0.66 ± 0.02	0.67 ± 0.02	+0.01	0.69 ± 0.08	0.65 ± 0.08	−0.04	0.77 ± 0.10	0.70 ± 0.09	−0.07	0.97 ± 0.01	**0.93** ± 0.03	−0.04
o3 mini	0.67 ± 0.06	0.69 ± 0.05	+0.02	0.65 ± 0.03	0.68 ± 0.02	+0.03	0.79 ± 0.02	0.77 ± 0.03	−0.02	0.84 ± 0.03	0.82 ± 0.04	−0.02	0.96 ± 0.02	0.95 ± 0.02	−0.01
Llama3 Med42 8B	0.20 ±0.09	0.19 ± 0.02	−0.01	0.61 ± 0.06	0.51 ± 0.04	−0.10	0.48 ± 0.10	0.41 ± 0.01	−0.07	0.53 ± 0.13	0.48 ± 0.04	−0.05	0.91 ± 0.02	0.86 ± 0.02	−0.05

Model performances varied with validation requirements. GPT-4o experienced a strong performance drop for Comprehensiveness with RAG, while Qwen 2.5 14B, DSR Llama 70B and o3 mini improved. However, GPT-4o remained the strongest model. $\Delta$*RAG is obtained as the difference of “without (w/o) RAG” and RAG. Highest scores per column are printed in bold*.

### System prompt specificity and test case structure affect model performance

**Fig. 3:**
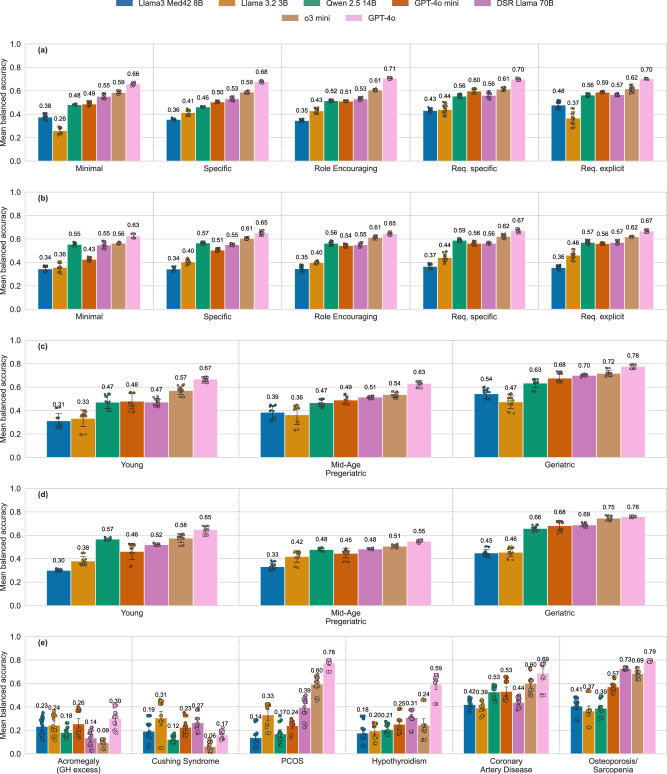
LLM mean balanced accuracy across various system prompts, age groups and diseases. **a** Overview of LLM performance across five system prompts without the application of RAG. Llama 3.2 3B, Qwen 2.5 14B but also GPT-4o mini and Llama3 Med42 8B exhibit a notable dependence on the system prompt in terms of response quality. **b** System-prompt-specific LLM performance with RAG. **c** LLM performance distribution across three different age groups without RAG. All models achieve significantly higher performance for geriatric individuals compared to the other two age groups (*P* < 0.001). **d** LLM performance distribution across three different age groups with RAG. **e** LLM performance distribution across diseases without RAG. LLMs show increasing scores in case of degenerative diseases. *The mean balanced accuracy is displayed above each bar. Error bars and individual data points are shown*. GH Growth hormone, PCOS Polycystic ovarian syndrome.

GPT-4o performed significantly better than the other models across all system prompts *(P* < *0.001)* and achieved high performance levels for even the least specific prompts (“Minimal”, “Specific”, Fig. 3a). With increasing specificity of the system prompt, medium-performing models (Qwen 2.5 14B, GPT-4o mini, DSR Llama 70B) improved by 0.02 to 0.18 in terms of balanced accuracy (at maximum, from 0.26 to 0.44). Llama3 Med42 8B showed its highest performance gains when using the most sophisticated prompt, “Req. explicit”. Across system prompts, top-performing models experienced insignificant performance declines with the application of RAG, while modest but significant improvements were observed for lower-performing models (e.g., Qwen 2.5 14B; *P* < *0.001* for “Minimal” and “Specific”, *P* = *0.01* for “Role Encouraging”; Fig. 3b). By contrast, the quality of Llama3 Med42’s responses significantly decreased for “Req. specific” and “Req. explicit”‘ when RAG was applied (*P* < *0.001*, Table 1). For further information on how the system prompts affected model accuracy across all requirements, please refer to Supplementary Tables 1–4 (Supplementary Section K). The vulnerability of the models to variations in backgrounds (short, verbose), profiles (paragraph-based, list-based), and distractors (with distractor, without distractor within a test case) was evaluated in an ablation study, in which all profile variations resulting from the components of a test item were tested. Vulnerability was highest for Llama 3.2 3B and Qwen 2.5 14B, with Llama 3.2 3B showing susceptibility to the injection of distractors. Overall, all other models showed only minor vulnerabilities (Supplementary Figs. 9 and 10 in Supplementary Section K).

### Accuracy of LLM responses correlates with the age of the user asking for advice

Mean balanced accuracy generally increased across age groups from young/mid-aged to geriatric (Fig. 3c, Table 2), see also Supplementary Tables 5 and 6 (Supplementary Section L); this was not affected by RAG (Fig. 3d, Table 2). GPT-4o again shows the highest mean balanced accuracy (*P* < 0.001), while Llama 3.2 3B and Llama3 Med42 8B again perform significantly worse than the other models, across all age groups (*P* < 0.001). The test items featured age-group-specific diseases, and LLMs performed better when faced with the widespread musculoskeletal and cardiovascular diseases in the “geriatric” age group, as compared to the less frequent hormonal diseases in the other groups (Fig. 3e).

**Table 2:** Mean balanced accuracy of models across different age groups without (w/o) and with RAG

Evaluated Models	Young			Mid-Age/Pregeriatric			Geriatric		
	w/o RAG	RAG	$\Delta$RAG	w/o RAG	RAG	$\Delta$RAG	w/o RAG	RAG	$\Delta$RAG
Llama 3.2 3B	0.33 ± 0.07	0.38 ± 0.04	+0.05	0.36 ± 0.08	0.42 ± 0.05	**+0.06**	0.47 ± 0.06	0.46 ± 0.04	−0.01
Qwen 2.5 14B	0.47 ± 0.06	0.57 ± 0.01	**+0.10**	0.47 ± 0.03	0.48 ± 0.01	+0.01	0.63 ± 0.04	0.66 ± 0.02	**+0.03**
DSR Llama 70B	0.47 ± 0.03	0.52 ± 0.01	+0.05	0.51 ± 0.01	0.48 ± 0.01	−0.03	0.70 ± 0.01	0.69 ± 0.02	−0.01
GPT-4o	**0.67** ± 0.02	**0.65** ± 0.03	−0.02	**0.63** ± 0.02	**0.55** ± 0.01	−0.08	**0.78** ± 0.02	**0.76** ± 0.01	−0.02
GPT-4o mini	0.48 ± 0.07	0.46 ± 0.07	−0.02	0.49 ± 0.03	0.45 ± 0.04	−0.04	0.68 ± 0.04	0.68 ± 0.04	±0
o3 mini	0.57 ± 0.04	0.58 ± 0.04	+0.01	0.54 ± 0.02	0.51 ± 0.01	−0.03	0.72 ± 0.03	0.75 ± 0.02	**+0.03**
Llama3 Med42 8B	0.31 ± 0.06	0.30 ± 0.01	−0.01	0.39 ± 0.06	0.33 ± 0.03	−0.06	0.54 ± 0.04	0.45 ± 0.03	−0.09

In both scenarios (w/o RAG and with RAG), all models achieve their highest scores for the ”geriatric” age group. *Highest scores per column are printed in bold*.

### Evaluation of interrater reliability between LLM-based judge and a human rater

**Fig. 4:**
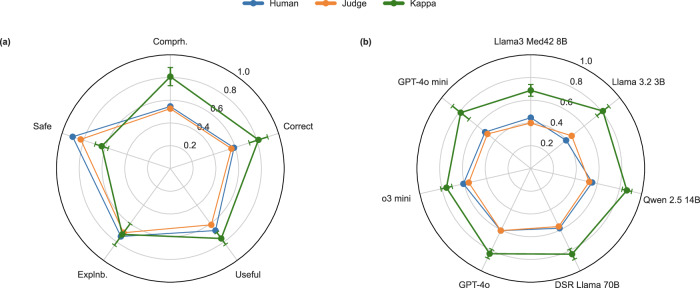
Alignment between human rater and LLM-based judge. **a** Mean balanced accuracies achieved by the models across all validation requirements, as assessed by the human rater and the LLM-based judge. **b** Overall accuracies per model. *Both subplots additionally illustrate Cohen’s kappa scores, which are used as a measure of alignment between the human rater and the LLM-based judge. Error bars indicate variability in alignment*.

We further examined the alignment between the judgments of a human rater (HJ) and GPT-4o mini as the LLM-as-a-judge using a sample of generated responses and their associated LLM judgments. The responses were sampled randomly and evaluated in a blinded fashion. This experiment was conducted to assess the validity of the LLM-as-a-Judge paradigm in our setting. With Cohen’s kappa scores ranging from 0.69 (Llama3 Med42 8B) to 0.87 (Qwen 2.5 14B) across models and from 0.63 (Safe) to 0.81 (Correct) across validation requirements, the results indicate consistently high alignment (Fig. 4; see “Models” in Methods).

## Discussion

By testing performance across multiple validation requirements using modular, physician-approved test items, we went beyond the exam-based assessment of LLMs in a reproducible and transparent manner, allowing for the assessment of free-text tasks. We evaluated proprietary and open-source LLMs using a benchmark specifically designed for evaluating intervention recommendations in the fields of geroscience and longevity medicine. Using the LLM-as-a-judge approach, our findings demonstrated that current LLMs must still be used with caution for any unsupervised medical intervention recommendations. Indeed, LLMs showed inconsistent accuracy across validation requirements, rendering benchmarks that measure single dimensions of model performance insufficient to capture the full complexity of heterogeneous and test-item-specific model capabilities. This demonstrated the complexity of judging LLM responses, justifying a detailed analysis by the automated judging approach described in Fig. 1. However, we note that automated judgment cannot systematically validate all testing dimensions for their alignment with human judgements; the only exception was correctness, in the scenario where the expert-provided binary ground truth was either matched or not by the response of the LLM (see Supplementary Fig. 4 in Supplementary Section H). Then again, human judgements are prone to heterogeneity, errors, and biases, and it remains for future research to analyze their correlation with judgements by LLM-as-a-judge.

Overall, open-source models tended to perform worse than proprietary models, and response quality of the latter was mostly considered sufficient, triggering positive verdicts by the LLM-as-a-judge in most cases, see Fig. 2. Intriguingly, Llama3 Med42 8B, which as a biomedically fine-tuned model would be expected to perform well, exhibited difficulties in generating responses that sufficiently met the validation requirements. A potential contributing factor may be a strong alignment to the fine-tuning corpus (overfitting to specific tasks) and, thus, reduced ability to generalize to new datasets. Open-source models struggled particularly in achieving sufficient comprehensiveness. Along these lines, a recent study found that around 90% of research papers criticized the lack of comprehensiveness (defined heterogeneously, yet in alignment with our definition) in LLM-generated medical responses^28^. However, while a lack of comprehensiveness may mean that LLM outputs fail to reveal knowledge important to the user, comprehensive responses may be less *comprehensible* (useful) by overwhelming the user. Moreover, a notable positive aspect was that all models exhibited a high “Consideration of Toxicity/Safety”, such that any lack of comprehensiveness does not tend to imply the recommendation of a harmful intervention. This may reflect an alignment of LLMs with common human values, presumably a consequence of Reinforcement Learning via Human Feedback (RLHF). Of note, the alignment between human rater and LLM was lowest for the safety requirement out of all requirements (Cohen’s kappa of 0.63). The primary responses received *higher* ratings for safety from the human evaluator, implying that the generally high safety score in the full benchmark could even be an underestimate. From an ethical perspective, safety is fundamental (reflecting the principle of non-maleficence), yet in our application domain, overly cautious model behavior may mean that no intervention is recommended – not even diet or exercise; this may not be in the interest of the user. Also, while comprehensive responses may pose cognitive challenges for users, a lack of comprehensiveness may harm informed decision-making and thus the principle of autonomy. Ethically, comprehensiveness must thus be balanced with comprehensibility; it cannot be neglected without compromising user empowerment^29,30^.

Many studies have already demonstrated that LLM responses can be highly dependent on prompt design and on the ordering of information within a prompt^31,32^, posing a risk in healthcare in particular. In our case, even small modifications in test case structure (e.g., increased verbosity) led to performance differences across prompt settings. However, LLMs demonstrated stability when exposed to irrelevant (distracting) statements, maintaining focus on the main query. This is a positive outcome, though the possibility remains that more complex distractions could affect performance. Generally, prompt sensitivity is not inherently a disadvantage; it can be beneficial when used intentionally for performance enhancement through prompt engineering. Our study found that instructive and advanced system prompts, which request specific and detailed reasoning by pointing out the validation requirements, improve performance by up to 0.18 in balanced accuracy for medium-performing models. Curiously, this improvement, predicted in ref. ^25^, was triggered by mere mentioning of the requirements, whereas quoting their explicit definition resulted in no additional gains (but compare the improvements by system prompt complexity for Llama 3.2 3B and GPT-4o mini, Fig. 3a). However, state-of-the-art commercial models such as GPT-4o and o3 mini already perform consistently well with simple prompts, showing only slight improvements when given additional instructions.

In our study, LLMs appeared to exhibit age-related performance bias^33^, which however may be induced by the differential incidence of diseases represented in the corresponding test cases. Indeed, our framework revealed that LLMs are more likely to correctly identify frequently observed degenerative diseases compared to rare hormonal conditions, demonstrating that the age bias may be explained at least in part by the age-associated prevalence of certain diseases, see Fig. 3c–e. RAG led to model-dependent increases or decreases in accuracy. This is interesting since RAG is typically used to mitigate knowledge gaps and improve response quality. The observed decline in accuracy under RAG, as also noted in GPT-4o, may be attributable to alignment of the training data with biomedical content. However, Llama3 Med42 8B also exhibits a notable performance reduction. Thus, another explanation could be that the introduction of non-relevant or low-utility content by RAG could dilute the effective signal and disrupt baseline model performance; this may also hold in more sophisticated models. Given the growing interest in clinically applicable RAG systems^34,35^, future research should explore how RAG-based applications affect different dimensions of model response quality, helping to determine which aspects of LLM performance are most influenced by this strategy. As a clear limitation, we applied only one frequently implemented flavor of RAG based on a database of papers relevant to longevity interventions.

There are general limitations to our study. Our benchmark started with queries synthesized for 25 fictional individuals, and use of real-world queries would have provided more authenticity at the expense of a much higher heterogeneity and a lack of patterns such as the ones used to investigate the role of the age group and the underlying disease. By generating 1000 test cases through modular variation, we mimicked some real-world diversity. We selected only 25 synthetically generated and annotated test items, because the development of the items, along with the associated references and ground truths, required substantial expert input and multiple rounds of refinement. We acknowledge that the small sample size may limit the generalizability of our findings beyond the test cases we investigated. Nevertheless, the modular structure of the 25 test items, in combination with various system prompts, resulted in numerous prompt variations per test item. By focusing on methodological advancements, our test procedure combines automated test generation with evaluation via the LLM-as-a-Judge paradigm. It operates without human assistance, thereby achieving an efficient use of expert time. In addition, the benchmark is designed to be easily extensible and adaptable for assessing future models. Moreover, the test items were designed to provide the LLMs with more comprehensive information than would typically be supplied in a standard user query, allowing the models to fully demonstrate their capabilities in generating personalized recommendations under conditions where all relevant data are readily accessible. Future work should examine scenarios with less complete input, and explore the added complexity of typical user-LLM dialogues.

Another limitation is the use of an LLM-as-a-Judge to evaluate tested LLMs, which may introduce model-specific biases, that is, the tendency of judgments to favor the responses from certain models rather than assessing them based on, e.g., a predefined metric. To mitigate this, we provided physician-validated ground truths to the LLM-as-a-judge. Despite conducting experiments that examined the alignment between a human rater and the LLM-based judge, which demonstrated high inter-rater reliability within our setting, it should be noted that we did not perform a comprehensive human evaluation of the full benchmark dataset. Thus, further studies are needed to assess the consistency of automated judgments, and also to compare these to human evaluations. Furthermore, while our study examined performance differences based on age and disease, it did not explore how other definitions of the age groups, swapping ages within test cases, or including a higher variety of diseases might influence LLM behavior. More elaborate item templates, e.g., by “symbolization”^32^, are left to future investigations. In addition, we focused on integrating five well-known longevity interventions, but have to acknowledge that this selection is not able to capture all available interventions. We focused on longevity interventions with enough evidence to form an expert opinion, which excludes many experimental and more recent interventions.

Popular medical and biomedical benchmarks, including MedQA, MedMCQA, MultiMedQA and the MIMIC datasets (including MIMIC-III^36^, MIMIC-IV-ED^37^, MIMIC-IV-ICD^38^) primarily assess LLM performance using multiple-choice question formats. While valuable, these approaches often fail to capture important nuances of model capabilities, such as personalization or robustness in open-ended tasks. Here, we developed a benchmark designed to evaluate LLMs across five validation requirements using modular, open-ended test items. These items focused on personalized intervention recommendations in geroscience and longevity medicine and were aligned with physician expertise through expert annotation. Our systematic and automated model evaluation approach enables testing LLMs in various medical domains. Future work could explore the extension of our framework to real-world clinical settings and continuous evaluation as models evolve. To facilitate this effort, the frameworks used and developed in this study are freely available and intended to be adapted and extended by other researchers for benchmarking models in diverse medical or other research contexts.

## Methods

### Benchmark dataset and test items/user prompts

We developed a benchmark of 25 test items assessing personalized LLM advice on longevity interventions and then tested the LLMs across the mentioned 5 validation requirements, as defined comprehensively in Supplementary Section A; in most evaluation scenarios, these requirements were given as an explicit guide to the LLM-as-a-judge. We emphasize that the test items comprise synthetically drafted medical profiles for benchmarking purposes; no real patient data was used.

One of us (HJ) drafted the test items along with the ground truths, which centered around expert commentaries with keywords, describing what is expected from the LLM response, such as the gains and caveats to consider. In this context, the keywords distill the core content of the expert commentaries and function as supplementary input for the LLM-as-a-Judge. Additionally, each query was designed so that a “Yes” or “No” response (binary ground truth) could be assigned, indicating whether an intervention is recommended or not, see Supplementary Section B.

Four domain experts (AH, BZ, CB, SF) reviewed the test items and ground truths in three rounds (“Benchmark Development” in Fig. 1). Initially, subsets of items were reviewed independently (“1st” [round of expert assessment] in Fig. 1), followed by a revision of the full benchmark in the second round (“2nd” [round of expert assessment] in Fig. 1). The test items were then structured into standardized modules: background information, biomarker profile, and the final binary question (“Yes” or “No”). To simulate diverse conversational scenarios, variations were created by rephrasing backgrounds and profiles into different formats (short or verbose backgrounds, paragraph-based or list-based profiles), with an additional “distracting statement” - placed at the end of a test case or not, to test the LLM’s robustness against irrelevant information (“Rephrasing” in Fig. 1). In the third round, all experts re-reviewed the full benchmark (“3rd” [round of expert assessment] in Fig. 1). The final structure of a test item is illustrated in Supplementary Fig. 1, while the development of this structure during the three-round expert review process is shown in Supplementary Fig. 2 (see Supplementary Section B).

During automated benchmarking (“Execution” in Fig. 1) eight different test cases were thus created from one test item’s modules and used as user prompts. Together with five different system prompts (see below), this modular approach enabled the automated generation of 8 * 5 * 25 = 1000 test cases from the 25 modular items. The structure of a finalized test case and its combinatorial assembly are illustrated in Supplementary Sections B and C. All 25 test items are listed in Supplementary Section D.

### Domain background and Retrieval-Augmented Generation (RAG)

The benchmarking data features clinical biomarker data from various individuals who wish to undertake one or a combination of the following longevity interventions: caloric restriction (*n* = 6), intermittent fasting (*n* = 4), exercise (*n* = 5), a combination of caloric restriction and exercise (*n* = 4), and the intake of supplements or drugs commonly associated with health effects. The latter are Epicatechin (*n* = 2), Fisetin (*n* = 1), Spermidine (*n* = 1), and Rapamycin (*n* = 2); see Supplementary Section E for background information. Furthermore, the individuals were categorized into the following age groups: young (20–39 years, *n* = *11*), mid-aged (40–60 years, *n* = 7), and elderly/geriatric (>60 years, *n* = 7). Five young and mid-aged profiles indicate the presence of the risk for an underlying hormonal disorder (hypothyroidism, cushing syndrome, acromegaly, and polycystic ovarian syndrome [PCOS]) for which longevity interventions should not be the primary recommendation. Additionally, for four “geriatric” profiles, the application of longevity interventions is contraindicated due to age-related musculoskeletal (osteoporosis and sarcopenia) or cardiovascular (coronary artery disease, *two cases*) diseases, along with their respective comorbidities. These diseases are noted, together with potential differential diagnoses, in the expert commentaries.

To test the effect of RAG on LLM response quality, we appended RAG-based data to the user prompts, for which a vector database was created using QDrant (https://qdrant.tech/), containing approximately 18 000 open-source scientific research papers with focus on the fields of geroscience and longevity medicine, see Supplementary Section F.

### System prompts

We defined five different system prompts with varying complexity that are automatically combined with the user prompts, where the information content of the instructions increases from “Minimal” towards “Requirements-explicit”. “Minimal” prompts the LLM to return, at the end of the answer, either “Yes” or “No”, stating whether the intervention is recommended or not. “Specific” adds that the query relates to longevity medicine, geroscience, aging research and geroprotection. “Role encouraging” additionally integrates a definition of the advisory role that the LLM is expected to assume. “Requirements-specific” further lists the five validation requirements the LLM should fulfill in its response, while “Requirements-explicit” additionally provides the definitions of these requirements. The instructions to the LLM-as-a-judge then included the test case, the response of the LLM being evaluated and the expert annotated ground truths, see Fig. 1, while the binary ground truth was added only in some evaluation scenarios when the LLM-as-a-judge had to evaluate the correctness of a model response; for more information on the system prompts see Supplementary Section G.

### Models

Proprietary LLMs available in February/March 2025 included GPT (Generative Pretrained Transformer) series models (OpenAI), specifically o3-mini (with “reasoning effort” set to medium), GPT-4o and GPT-4o mini, while open-source models selected were Llama 3.2 3B (by Meta)^39^, Qwen 2.5 14B and DeepSeek R1 Distill Llama 70B (DSR Llama 70B for short), which is built based on Llama 3.3 70B. All models were accessed via the appropriate APIs (OpenAI API, Groq, LMStudio). Considering the biomedical orientation of our benchmark, it was of particular interest to evaluate how biomedical fine-tuned models perform in the test. We selected Llama3 Med42 8B^40^, an 8 billion-parameter domain-tuned model trained on biomedical literature and datasets, and first evaluated it alongside OpenBioLLM3 8B. Prior to our benchmark, both models thus underwent a pre-assessment using the AMEGA Benchmark^23^, which is oriented toward clinical treatment recommendations. We integrated all 20 AMEGA cases, along with the 135 questions and their corresponding ground truths, into our paradigm, and executed the AMEGA Benchmark on the two biomedical models and the 6 models we already introduced. Llama3 Med42 8B (balanced accuracy: 0.63) outperformed OpenBioLLM3 8B (0.36) but both models performed worse than open-source and proprietary models (e.g., Qwen 2.5 14B: 0.82, GPT-4o: 0.89). Llama3 Med42 8B was thus chosen for inclusion in our benchmark. For more information we refer to Supplementary Fig. 3 (Supplementary Section H).

Llama 3.2 3B, Qwen 2.5 14B, DSR Llama 70B, GPT-4o mini, o3 mini and GPT-4o were evaluated in the time period February-March 2025. Llama3 Med42 8B and OpenBioLLM3 8B were tested in August 2025. Except for o3-mini, all models were tested using greedy decoding (temperature 0). o3-mini was used with default temperature settings (temperature = 1), as OpenAI offered this model only through an API program which does not allow for custom adjustments of temperature.

To further elucidate the robustness of the judgements within the final testing environment for the main benchmark, both GPT-4o mini and GPT-4o were used to assess correctness in two evaluation settings: one when given the binary ground truth (standard setting) and one without. We selected GPT-4o mini as the final LLM-as-a-Judge for our experiments because GPT-4o mini’s judgments showed higher alignment with the ground truth in both evaluation settings, while a comparative analysis across all validation requirements revealed that both models showed high interrater reliabilities for Correctness. For further information please refer to Supplementary Figs. 4 and 5 (Supplementary Section H).

To assess the agreement between LLM-based judgments and human evaluation, we conducted an alignment check using randomly sampled test item variations. Model responses were blindly evaluated by a human rater (HJ) across all validation requirements, resulting in a total of 1000 individual judgments. These human ratings were then compared with those of GPT-4o mini (Fig. 4).

### Performance evaluation

The BioChatter framework^27,41^ was used for automated performance assessment, including the collection of model outputs providing these together with the ground truths to the LLM-as-a-Judge; this was done *n* = *4* times, and repeated with RAG for the responding (not the judging) LLM. For each response, the judgement was conducted *two* times, returning a verdict (score) in binary format, e.g., “comprehensive” or “not comprehensive” for comprehensiveness; this resulted in 280000 verdicts. Then, the verdicts were transformed to binary numeric values consisting of 0 (failure, e.g., “not comprehensive”) and 1 (success, e.g., “comprehensive”). Judgement was performed twice, and 1% of all judgements resulted in an intermediate score of 0.5. These were binarized as “0” (failure). For further information on the judgement procedure, we refer to Fig. 1, and Supplementary Sections I (structure of the judgement framework) and J (example interaction between researcher and framework).

### Statistical analysis

Statistical analyses were conducted using Pingouin (0.5.5)^42^, Scikit-learn (1.6.1)^43^ and SciPy (1.15.2)^44^ in Python (version 3.11.2). The mean balanced accuracies of the models were determined based on the LLM judge’s verdicts and compared across models. To evaluate overall differences in balanced accuracy among all models for each validation requirement and system prompt, we applied Cochran’s Q test. Pairwise differences in model accuracies were assessed using McNemar’s test. To examine differences in grouped model accuracy across age groups, we used the Chi-square test. A *p*-value of *P* < *0.05* was considered statistically significant. All *p*-values were Bonferroni-corrected to account for multiple comparisons. The performance of the models is measured as their balanced accuracy scores in addressing the evaluation criteria, i.e., the validation requirements. Interrater reliabilities were evaluated by calculating Cohen’s kappa.

## Supplementary information

Manuscript_Supplement_3

## Data Availability

The benchmarking data are openly available on GitHub, at https://github.com/biocypher/biochatter.

## Code availability

The code for this study is implemented as a part of https://github.com/biocypher/biochatter. The repository is additionally archived by Zenodo integration at https://zenodo.org/records/14775193.
